# The Thermo-Mechanical Coupling Effect in Selective Laser Melting of Aluminum Alloy Powder

**DOI:** 10.3390/ma14071673

**Published:** 2021-03-29

**Authors:** Xianyin Duan, Xinyue Chen, Kunpeng Zhu, Tao Long, Shiyang Huang, Fuh Y H Jerry

**Affiliations:** 1 Key Laboratory of Metallurgical Equipment and Control Technology, Wuhan University of Science and Technology, Ministry of Education, Wuhan 430000, China; xyduan@wust.edu.cn (X.D.); chen_cxyck@163.com (X.C.); l273321360@163.com (T.L.); hsy960411@163.com (S.H.); 2 Institute of Advanced Manufacturing Technology, Chinese Academy of Sciences, Changzhou 213000, China; 3 Suzhou Research Institute, National University of Singapore (NUS), Suzhou 234000, China; jerry.fuh@nus.edu.sg

**Keywords:** transient temperature field, selective laser melting, finite element, stress and deformation

## Abstract

In the selective laser melting process, metal powder melted by the laser heat source generates large instantaneous energy, resulting in transient high temperature and complex stress distribution. Different temperature gradients and anisotropy finally determine the microstructure after melting and affect the build quality and mechanical properties as a result. It is important to monitor and investigate the temperature and stress distribution evolution. Due to the difficulties in online monitoring, finite element methods (FEM) are used to simulate and predict the building process in real time. In this paper, a thermo-mechanical coupled FEM model is developed to predict the thermal behaviors of the melt pool by using Gaussian moving heat source. The model could simulate the shapes of the melt pool, distributions of temperature and stress under different process parameters through FEM. The influences of scanning speed, laser power, and spot diameter on the distribution of the melt pool temperature and stress are investigated in the SLM process of Al6063, which is widely applied in aerospace, transportation, construction and other fields due to its good corrosion resistance, sufficient strength and excellent process performance. Based on transient analysis, the relationships are identified among these process parameters and the melt pool morphology, distribution of temperature and thermal stress. It is shown that the maximum temperature at the center point of the scanning tracks will gradually increase with the increment of laser power under the effect of thermal accumulation and heat conduction, as the preceded scanning will preheat the subsequent scanning tracks. It is recommended that the parameters with optimized laser power (*P* = 175–200 W), scanning speed (*v* = 200–300 mm/s) and spot diameter (*D* = 0.1–0.15 mm) of aluminum alloy powder can produce a high building quality of the SLM parts under the pre-set conditions.

## 1. Introduction

Compared to traditional production methods such as welding, casting, and machining, selective laser melting (SLM) enables the one-time forming of complex-shaped metal parts with high-performance and high-density. Therefore, selective laser melting is widely used in advanced fields such as molding, aerospace, biomedicine, etc. It has many advantages over traditional machining in building precision parts. However, in the process of selective laser melting, the concentration of the laser heat source increased the surface temperature above the material liquidus. The laser heat source entered the powder surface layer by heat transferring and absorbing [1,2,3], resulting in a very heterogeneous temperature and stress distribution. The selective laser melting was accompanied by extreme changes in temperature, stress, splash, and melt pool morphology, which resulted in material anisotropy. Such layer-by-layer melt building processes had many specific defects in the microstructure, macrostructure, and mechanical properties of the formed parts.

In recent years, many scholars have carried out research on monitoring the melt pool morphology or simulating the temperature and stress field of the building process. In the melt pool morphology monitoring, research was usually conducted through online monitoring experiments along with SLM building, and then the feature extraction and classification to identify the defects under different process parameters. Zhang et al. [4] proposed an image acquisition system based on high-speed cameras. Artificial intelligence methods were used to classify and identify melt pools, plumes, and splashes. Fuh et al. [5] designed a new image processing method using Kalman filter tracking technology to accurately locate the melt pool, whose image and size monitoring could well reflect the building state. Ye et al. [6] used a near-infrared (NIR) camera to monitor the formation of a single track on 304L stainless steel powder. The collection of images found that the characteristics of plume and splash in SLM under different forming parameters are different. Domrös et al. [7], Grasso et al. [8], and Ali et al. [9] used the multi-sensor coaxial system to monitor the plume and pores in the SLM online. The instability and special defects produced in the SLM process were observed to improve the forming quality by recognizing and controlling the defects. These studies pushed forward the online monitoring methods to identify the defects, by which the quality of building parts could be improved effectively. However, the current progress of online monitoring relied heavily on established monitoring systems which need high-performance sensors and complicated algorithms for data collection and processing. In particular, there was almost no solution to acquiring in real-time the temperature and stress field of the whole melt pool. 

Real-time temperature field simulation would be affected by many factors, such as the process parameters, preheating temperature, chamber temperature, etc., which ultimately affected the melt pool shapes and quality. The FEM was widely used in the temperature field simulation whose main task was to analyze the temperature distribution and temperature gradient changes and to predict defects. Most current studies have focused on the effect of melt pool heat convection on the temperature distribution by FEM. 

Kolossov [10] used FEM based on continuum theory to simulate the temperature evolution and parts building, which was affected by changing the material thermal properties. Yadava et al. [11] established a transient finite element thermal model of titanium alloy to calculate the temperature distribution and temperature gradient along the X- and Y-axis under different process parameters in a single metal layer. Cao et al. [12] studied the effect of particle size distribution, compactness, and layer thickness, respectively, on the formation of a single track in SLM through finite element simulation. Ding et al. [13], Li et al [14], Chua et al. [15], and Huang et al. [16] numerically simulated the heat transfer and temperature field in the SLM process. Ding et al. [13] revealed the significant effects of point pitch on the temperature field using spot exposure scanning. Li et al [14] reported the change rule of the temperature field using the finite difference method by the heat transfer equation. Chua et al. [15] incorporated the conversion from powder to solid into the proposed finite element model. Finally, a study by Huang et al. [16] showed that a higher maximum temperature will lead to larger melt pools. These researches studied the influence of different parameters and material properties on the temperature distribution through establishing three-dimensional finite element simulation using heat transfer and hydromechanics. However, only the temperature field could not exactly reflect the final building state and quality because the stress field also affects the building quality to a great extent. It was necessary to add the stress distribution in the finite element simulation and analysis during the building process due to the great influence of stress.

When the laser scanned according to a certain strategy, the metal powders melted into a comet-shaped melt pool which would induce a large temperature gradient. Simultaneously, the temperature gradient would cause considerable thermal stress inside the powder layer and a further serious deformation of parts. The deformation could bring extreme challenges to the quality control of the parts. However, it was difficult to monitor the stress during the SLM process as the heat transfer occurred inside the tiny melt pool. Most scholars simulated the real-time stress using FEM to achieve predictions without experimentation. Matsumoto et al. [17] studied the possibility of cracking and the location of cracks in the building layer by simulating the stress distribution using the heat conduction theory through FEM. Tawfik et al. [18] drew the conclusion after simulating the deposition process through FEM that the influence of laser scanning speed on deformation would become more obvious with subsequent layer stacking. Liu et al. [19] simulated the parts deformation in the SLM of AlSl304 stainless steel under the same material matrix using the finite element method and reported that large tensile stress was found in the deposited material when the workpiece was cooled. Cheng et al. [20] studied the influences of different scanning strategies on part deformation and internal stress in In718 multilayer SLM, which indicated that the interface between the substrate and the deposited layer would generate high stress. The above researchers simulated the stress distribution during the building process using FEM combined with theoretical calculations to predict and analyze the possible defects such as cracks and warpage, which provided good support and reference for related research. 

Based on the above literature study, many scholars have performed various finite element simulations, focusing on the simulation of melt pool shapes, temperature, stress, and deformation in the building process. However, there are few reports of simulation considering the temperature field and stress field simultaneously, which could not exactly reflect the final building state and quality. It was necessary to simulate the temperature and stress field coupled with a thermo-mechanical effect.

This paper proposed a transient thermo-mechanical coupled finite element model to predict the thermal behavior in selective laser melting of aluminum alloy powder. Based on this model, research on the influence of process parameters including laser power, scanning speed and spot diameter was carried out. Then, the relationship between process parameters and the melt dimensions, temperature distribution, and thermal stress were established. The simulation results showed that different process parameters had different influences on the building process under thermal and mechanical coupling. An increase in scan times would produce a heat accumulation effect, which led to the rise of the building region temperature and melt pool size. The rise of laser power would make the powder particles absorb more heat and would result in a higher temperature gradient and thermal stress deformation, while the increase of the scanning speed and the spot diameter would produce opposite results. This study provided theoretical support for research on the evolution rules of SLM thermal behavior especially when the temperature and stress were difficult to monitor online.

## 2. The Thermo-Mechanical Coupling Effect by Powder Bed Finite Element Modeling

### 2.1. Establishment of a Finite Element Model and its Meshing

The SLM is a powder bed fusion process in which a high-temperature laser is used to melt a metal powder layer so that the powder is melted from particles into a liquid, and then solidified from liquid to solid. The process is accompanied by a sudden change in temperature. At the same time, the temperature change causes the expansion or shrinkage of the built part. Thermal stress and strain occur when the expansion or shrinkage of the material is restricted. FEM is a continuous function that uses the discrete solution of the whole area into small finite elements instead of the entire solution area. The formation of a micro-melt pool is not convenient for experimental research. According to the changing regular pattern of temperature and stress in the building process, this paper uses FEM to set the stress conditions on the basis of the temperature model and selects the thermal-mechanical coupling type for simulation.

**Figure 1:**
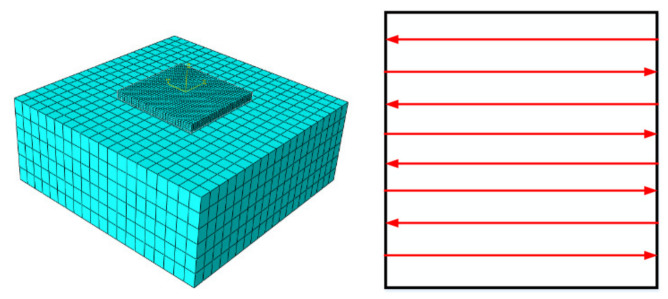
Finite element grid division and scanning strategy.

The SLM finite element grid division was carried out as shown in Figure 1, followed by the scanning strategy setting. The finite element model consists of two parts: 1045 steel as the substrate material at the bottom and Al6063 as the built layers on the top.

The size of the substrate and built layers are, respectively, 1.5 mm × 1.5 mm × 0.6 mm and 0.6 mm × 0.6 mm × 0.05 mm. About the size of the cell grid, the substrate is set to 0.05 mm × 0.05 mm × 0.05 mm, and the built layers are set to one-fifth of the substrate to make the grid of the built layers loaded with a moving heat source more refined. Considering the thermo-mechanical coupling analysis, the grid type is divided by the cathedral eight-node C3D8RT type.

### 2.2. Assignment of Material Thermal Properties

During the selective laser melting process, material converts from a powder to liquid state and finally to a solid state. In this process, the temperature varies closely with the conversion of the material state. Consequently, many thermal property parameters, such as thermal conductivity and the specific heat capacity of materials, will vary greatly with temperature. For instance, the thermal conductivity of the built part is several dozen times larger than the powder [21]. Therefore, it is significant to determine the material thermal parameters at different temperatures.

The thermal properties of the metal powder (Al6063) and the substrate material (1045 steel) summarized by the reference and material library are shown in Table 1.

The latent heat of the phase change, another thermal characteristic in addition to the above which occurs during phase transition, is the heat absorbed or released by a unit mass of powder from one phase to another among solid, liquid, and gas under an isothermal-isobaric environment. 

The ambient temperature varies along with the building process, which leads to the change of the phase properties. Therefore, the latent heat of the phase change needs to be taken into account in the FEM simulation.

Usually, the latent heat of the phase change is obtained by the equivalent specific heat capacity method [24]. Using this method, the phase transition region of Al6063 could be produced and is generally 615 to 655 °C, and maximum latent heat is 390,000 J/Kg.

### 2.3. Determination of Initial and Boundary Conditions

In the study of transient thermo-mechanical coupling in the SLM process, the laser heat source model has a relatively large influence on its calculation and analysis. If the loaded heat source is not suitable, it will cause a large gap between the simulation result and the actual result. In most situations, the Gaussian surface heat source [20] is chosen as the initial condition of the laser heat source. This heat source is more in line with the single laser beam heat distribution of the powder bed in the actual building process of SLM. Thus, this study adopts the Gaussian surface heat source, whose model can be indicated as

(1) $$q\left(r\right)=\frac{3\eta P}{\pi R^{2}}e^{\left(-\frac{3r^{2}}{R^{2}}\right)}$$

where *P* is the laser power; *η* is the material laser energy absorption rate, which is set to 0.1; *R* is the radius of the laser heat source; and *r* is the distance from any point on the built part to the laser spot center.

The heat source, whose movement is realized through embedded time, is loaded by the DFLUX subroutine programmed in Fortran. 

The Al6063 powder melts rapidly under laser irradiation and cools to a solid permanently during the process of SLM. At the same time, statue changes lead to the variation of material thermal conductivity and specific heat capacity, which, as mentioned above, will change with temperature. Under the effect of thermo-mechanical coupling, uneven temperature changes will cause the high temperature to expand, but the low temperature area will remain unchanged. At this time, uneven constraints will cause stress and deformation to occur. Temperature and stress are defined as nonlinear transient heat conduction and thermal deformation problems. Therefore, the setting of boundary conditions mainly considers heat transfer.

The heat transfer mechanism includes heat radiation, heat conduction and heat convection. According to Fourier’s law and energy conservation law, the heat transfer equation [25] can be obtained as

(2) $$\lambda \left(\frac{\partial^{2}T}{\partial x^{2}}+\frac{\partial^{2}T}{\partial y^{2}}+\frac{\partial^{2}T}{\partial z^{2}}\right)+Q=\rho C_{p}\frac{\partial T}{\partial t}$$

where *λ* is the powder thermal conductivity of Al6063; *T* is the temperature at time *t*; *Q* is the latent heat; *ρ* is the powder density; and *C_p_* is the specific heat capacity. 

The following three boundary conditions are used in the finite element modeling.

The first boundary condition is the initial temperature distribution of mainly the substrate and the powder.

(3) $$T_{0}=T\left(x,y,z,0\right)$$

where *T*_0_ is the initial temperature.

The second boundary condition is the input of the heat source model.

(4) $$k_{x}\frac{\partial T}{\partial x}n_{x}+k_{y}\frac{\partial T}{\partial y}n_{y}+k_{z}\frac{\partial T}{\partial z}n_{z}=q\left(r\right)$$

where *k_x_*, *k_y_*, and *k_z_* are the material heat transfer coefficients (W/m·K); and *n_x_*, *n_y_*, and *n_z_* are the direction cosines of the outward normal to the model surface.

The third boundary condition is heat loss, mainly containing heat convection and radiation to set the diffusion coefficient between the built layer and substrate.

(5) $$k_{x}\frac{\partial T}{\partial x}n_{x}+k_{y}\frac{\partial T}{\partial y}n_{y}+k_{z}\frac{\partial T}{\partial z}n_{z}=h\left(T_{s}-T_{m}\right)+\sigma \varepsilon \left(T_{s}{}^{4}-T_{e}{}^{4}\right)$$

where *h* is the convective heat transfer coefficient; *T_s_* is the temperature at the surface of the model; *T_m_* is the temperature of the heat transfer fluid in the environment; *σ* is the Stefan–Boltzman constant; *ε* is the radiation heat transfer coefficient; and *T_e_* is the temperature of the external environment.

According to the physical and chemical phenomena in the thermo-mechanical coupling of the SLM building process, the laser heat source generates a large amount of heat after melting the powder, so the effects of thermal diffusion, thermal radiation, and thermal convection need to be considered. Different heat conduction and convection are generated from the metal powder, the liquid metal, and the solid building layer. Therefore, the third boundary condition, i.e., Equation (5), is chosen to set the coefficients of the built layer. Among them, the thermal correlation coefficient can be selected according to the literature.

After the temperature boundary conditions are loaded, it is necessary to set constraints between the built part and the substrate according to the actual building situation. The substrate and the bottom of the built part are fixed. The yield strength of the powder material once fixed will decrease with the increase of temperature, which leads to deformation. The stress field of SLM is usually nonlinear, and the evolution of stress is very complicated. It is necessary to use the yield criterion, flow criterion, and strengthening criterion for theoretical calculation. The establishment of the thermo-mechanical coupling model will be realized only considering heat transfer, radiation, heat dissipation and restraint settings of the process.

## 3. Result and Discussion

Many methods can control the SLM’s complicated building process, whose heat transfer process occurs not only between gas, liquid, and solid states, but also contains interaction among photons, electrons, and plasma. There are many ways to control the SLM molding process by changing individual or overall process parameters.

The volumetric energy density equation [26] can be used to control the process parameters, which describes the average energy per volume of material in the SLM process.

(6) $$E_{v}=\frac{P}{v\cdot h\cdot t}$$

where *P* is the laser power, *v* is the scanning speed, *h* is the hatch spacing, and *t* is the powder layer thickness. The process parameters can be divided into pre-process (the correlation parameters in the above equation), in-process, and post-process. 

Many simulation studies have been carried out on the effects of these parameters to optimize materials’ anisotropy, process defects, and thermal stress. Meanwhile, some process parameters will also influence the temperature, stress, and quality of the built part, such as the spot diameter *D*, initial temperature *T_0_*, etc.

**Figure 2:**
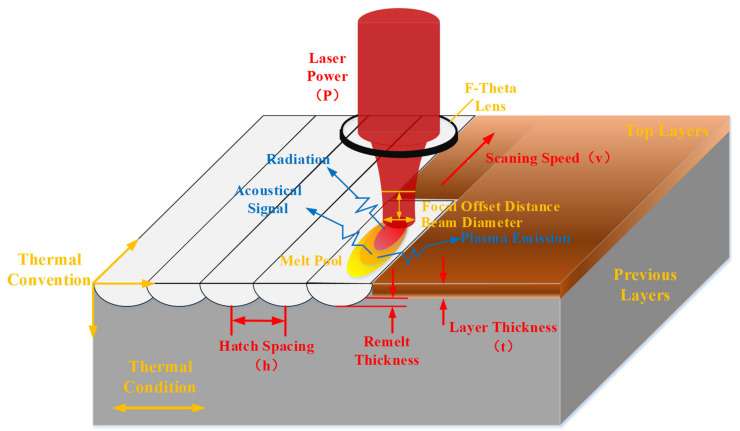
Thermal behavior in selective laser melting.

Figure 2 shows the thermal behavior and process parameters that can be changed in SLM. Several heat dissipation mechanisms appear in Figure 2 including plasma emission, phase change, heat convection, radiation, and conduction. Sometimes, particle splashing will directly remove heat from the system.

The process parameters, such as the laser power, scanning speed, and spot diameter, selected for the simulation of temperature and stress distribution in this paper, are shown in Table 2. The number of scanning is two with a zigzag scanning strategy in finite simulation.

### 3.1. Basic Characteristics of the Formed Melt Pool

**Figure 3:**
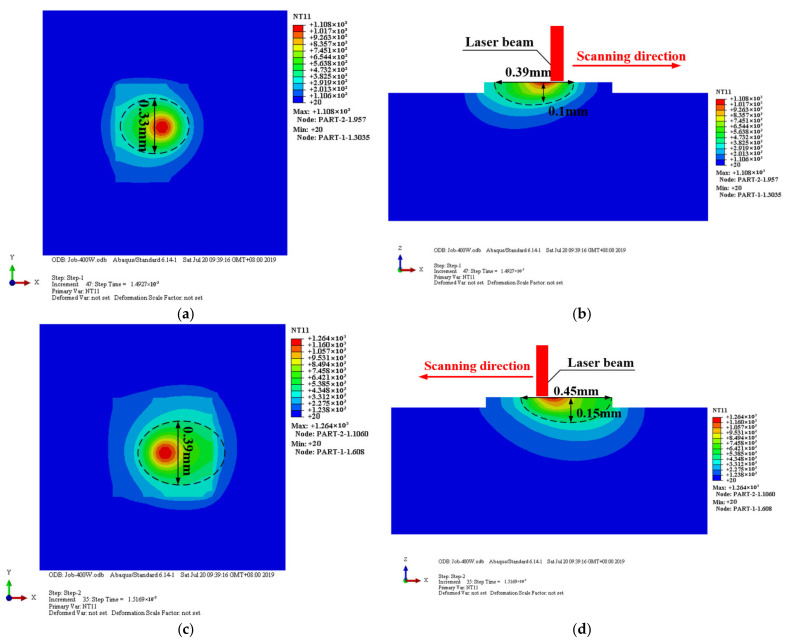
Temperature distribution at laser power *P* = 200 W, scanning speed *v* = 200 mm/s. (**a**) Surface temperature distribution at the first scanning midpoint. (**b**) Cross-section temperature distribution at the first scanning midpoint. (**c**) Surface temperature distribution at the second scanning midpoint. (**d**) Cross-section temperature distribution at the second scanning midpoint.

Figure 3 shows the temperature distribution of the midpoint powder layer (top view and vertical cross-section) when the laser power *P* = 200 W, scan speed *v* = 200 mm/s, scan pitch *s* = 0.07 mm, and spot diameter *D* = 0.2 mm. The strategy of the scanning is that the first laser beam passes through the powder layer from left to right, and the second passes from right to left.

The ellipse drawn by the dotted line in Figure 3 represents the Al6063 melting line (650 °C). The temperature inside the dotted line is higher than the Al6063 melting point, which can form a small melt pool. The melt pool after melting is mainly divided into three regions: the matrix, the fusion zone, and the heat-affected zone (HAZ), which are described with different colors in Figure 3. A higher temperature fusion zone above the melting line forms a small round red spot. The heat affected zone temperature around the melting line and is mostly elliptical. The matrix zone does not reach the melting line.

When the first laser beam scans through the center, the melt pool temperature drops from 1108 °C to 653 °C. The width, length, and depth of the melt pool are about 0.33 mm, 0.39 mm, and 0.1 mm, respectively. A time goes by, and the laser beam scans through the second track center, the temperature drops from 1264 °C to 745 °C. The width (0.39 mm), length (0.45 mm), and depth (0.15 mm) of the melt pool increase by 15%, 13%, and 33% respectively, compared to the first track.

It can be seen from Figure 3 that the temperature field is more densely distributed at the front of the melt pool than the end because the material transition from the powder to solid causes the thermal conductivity to increase, which is a benefit to the heat transfer.

According to the temperature nephogram during the SLM, the temperature and size of the melt pool gradually increase with the scanning times. The heat from the first track is diffused and generates a heat accumulation effect, which has an influence on the latter scanning. 

The heat dissipated by heat conduction is generally higher than heat convection and heat radiation during the building process. With the time of scans increasing, the ability to dissipate heat through heat conduction is impaired and reduced [27]. This is the reason why the melt pool temperature and the size increase.

The formation of the molten pool is affected by the flow of liquid metal during the building process. When the laser power is too low or the scanning speed is too fast, holes are easily formed inside the metal powder. At this time, the liquid molten pool flows from the center to the edge of the molten pool under the Marangoni effect, which pushed the spheroidized particles generated during the building process to the surface or edge of the building layer.

### 3.2. Influence of Process Parameters on the Distribution of Temperature and Stress

**Figure 4:**
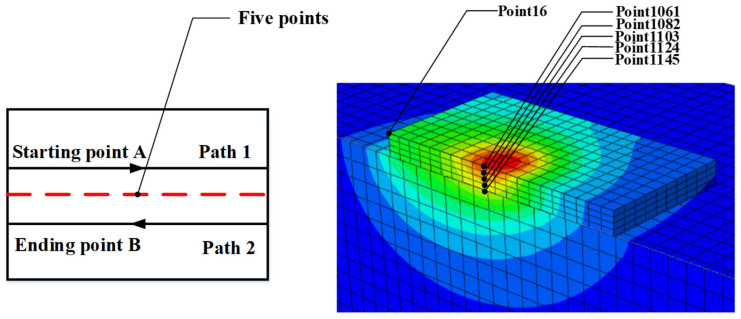
Node and center line selection.

The middle building area of the two laser paths (from A to B) is selected to study the simulation of the temperature distribution and stress deformation by changing the laser power, scanning speed and spot diameter in this paper. Five points on the midline are selected for data extraction after the simulation. Figure 4 shows the top view scanned by two lasers and the location of the selected points in the three-dimensional model. The simulation results are as follows.

#### 3.2.1. Temperature Distribution under Different Laser Powers

**Figure 5:**
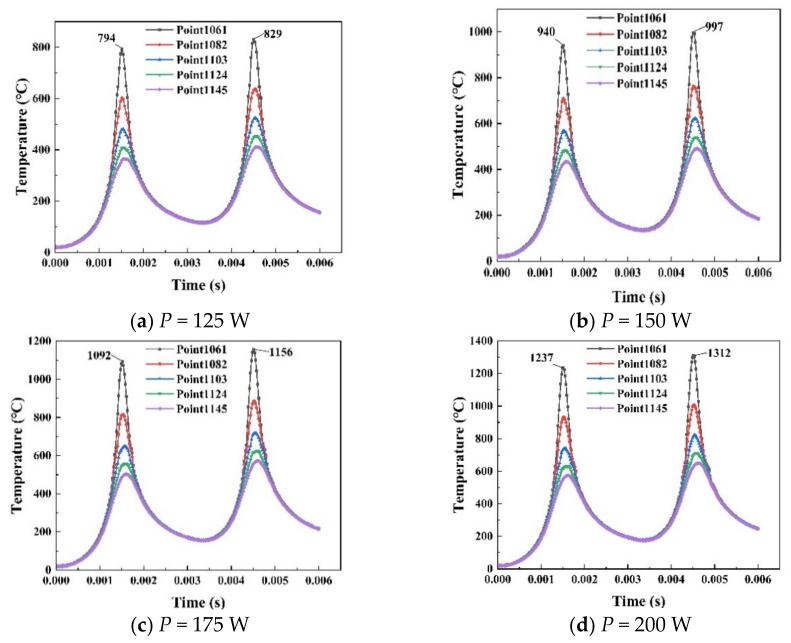
Midpoint temperature distribution curve of two scanning tracks with different powers (**a**) *P* = 125 W (**b**) *P* = 150 W (**c**) *P* = 175 W (**d**) *P* = 200 W.

The midpoint temperature curves with different laser powers (scanning speed is 200 mm/s, spot diameter is 0.1 mm) have been shown in Figure 5. The time of laser from the first track to second is 0.006 s. Two peaks appear in Figure 5 representing the temperature distribution of the heat source passing through the midpoint twice.

The temperature gradually rises as the laser power increases. When the laser power *P* = 125 W, the peak temperatures were 794 °C and 829 °C. When the laser power *P* = 150 W, the peak temperatures were 940 °C and 997 °C. When the laser power *P* = 175 W, the peak temperatures were 1092 °C and 1156 °C. When the laser power *P* = 200 W, the peak temperatures were 1237 °C and 1312 °C.

The temperature distribution of different points shows different rules. For example, the temperature rises when the heat source approaches the node. The powder layer absorbs less heat when the laser power is too small (*P* = 125 W). Points except Node 1061 are not completely melted when the first track passes, which may lead to insufficient powder layer melting. The defect is formed by a low overlap between the layers and finally affects the building quality. The penetration ability of high laser power will fully melt the material powder layer and lead to a good combination between layers, which builds a relative density material. Simultaneously, the laser power should not be too high. Higher laser power will evaporate the material and reduce the amount of powder in the melt pool, resulting in a lower density built molten track. When laser scanning the next pass, there is not enough powder to guarantee the quality of the melt pool.

**Figure 6:**
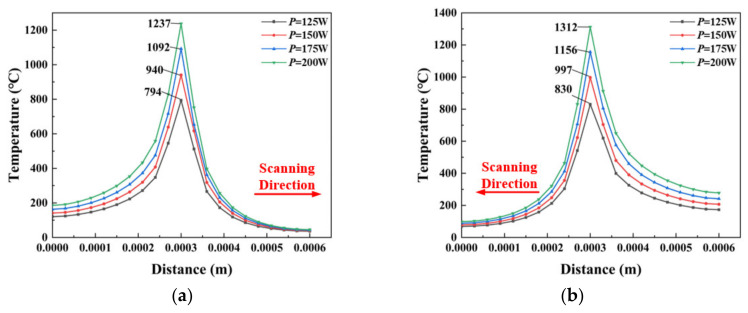
The temperature distribution of the laser heat source along the scanning direction under different powers at scan centers. (**a**) temperature distribution at *T* = 0.0015 s (**b**) temperature distribution at *T* = 0.0045 s.

Figure 6 shows the temperature distribution of the heat source along the scanning direction as it passes through the center of laser scanning in 0.0015 s and 0.0045 s with different powers. As is shown, the temperature rapidly rises to the peak when the heat source gradually approaches the midpoint. In contrast, the temperature drops rapidly. This is mainly because the release of solid latent heat and the liquid transforms into a solid. 

The peak temperature of the first laser scans is 1237 °C, and it reaches 1312 °C in the second. The temperature rises obviously when passing through the midpoint of the next track because of the heat accumulation of preheating in the material during the first laser scanning, but the preheating region is limited.

#### 3.2.2. Stress Distribution and Deformation under Different Laser Power

It can be seen from the simulation results that the difference in laser power will have a direct impact on the temperature distribution, which will indirectly affect the quality of building parts and cause some defects.

With the increase of laser power, the energy, which is input and transmitted into the substrate, increases simultaneously. The larger energy melt pool leads to an increase in the overlap rate, a decrease in porosity, and the material is fully melted. When the energy is too high, an expansion effect occurs inside the material, which will generate thermal stress. Local tensile stress will be generated at both ends of the building part, and local compressive stress will be generated in the middle. The thermal stress can be accumulated layer by layer by increasing the number of scans, and the building part will occur warping deformation. 

Conversely, the temperature and energy are correspondingly reduced when the laser power is lower. Adjacent melt pools cannot be fully dissolved due to low lap rates, resulting in pores. Pore formation will evolve into a spheroidization effect, which reduces the density of the built parts. The surface quality of the built part is affected by absorbing less heat and energy density. Compared to high laser power, low laser power has more defects for built parts.

**Figure 7:**
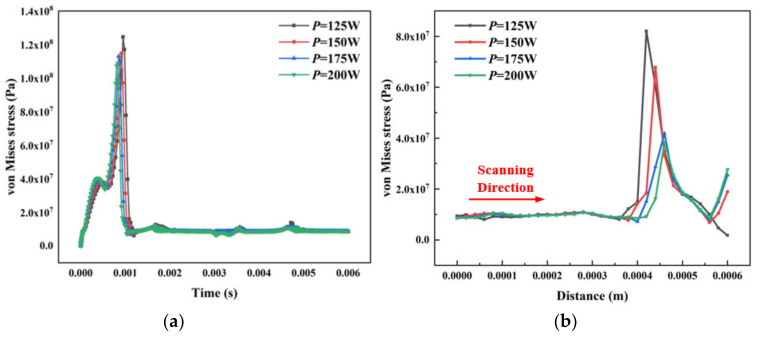
(**a**) Transient von Mises stress distribution through the scanning process under different laser powers. (**b**) The von Mises stress distribution at different laser powers in the same time.

Thermal coupling analysis is proposed to obtain von Mises stress and deformation of a point under different laser powers. The variation of the coupling force and displacement are studied based on building thermal behavior. Figure 7a shows transient von Mises stress distribution through the scanning process for point NT16 (shown in Figure 4) under different laser powers. It can be seen that stress increases gradually at the beginning. When *T* = 0.0005 s, stress suddenly drops and then rises linearly before *T* = 0.001 s. The stress value reaches maximum peak value at *T* = 0.001 s and reaches a stable state with little fluctuation. 

When the point is initially loaded with a heat source, the material changes from the metal powder to melting state due to a sudden increase in temperature, resulting in an extreme change in stress distribution. The stress is slightly reduced when converted into liquid because the heat conduction of solid is faster than liquid. When the liquid absorbs heat, the temperature and stress increase. When the heat source gradually moves away from the node, the temperature will remain within a range because the material cools down slowly after increasing with the small size of the simulation. The stress value will not fluctuate significantly with the change of temperature gradient. A small change in the material temperature gradient results in little fluctuation in stress.

Figure 7b shows the stress distribution at different laser powers in the same time. It is clear that the stress decreases as the laser power increases. The material can absorb more heat energy and most metal powder is fully melted and converted to liquid when the laser power increases. The phase composition in the melt pool is sufficiently combined, which reduces thermal stress. Low laser power causes the powder to melt insufficiently. The anisotropy between unmelted powder and liquid metal causes large transient stress deformation inside the material.

**Figure 8:**
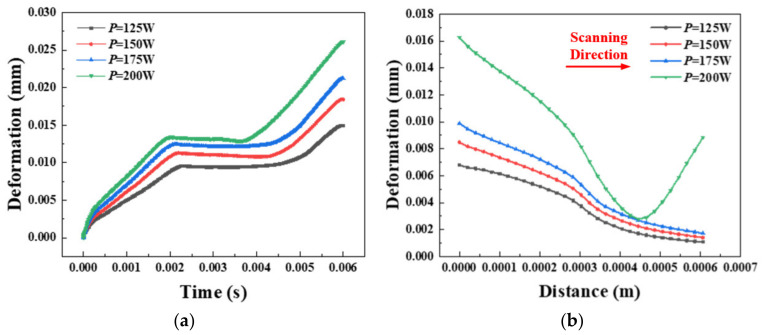
(**a**) Displacement deformation at different powers in the initial point of the midline. (**b**) Displacement deformation distribution under different powers at the same time.

Figure 8 shows the point displacement and the amount of transient deformation under different laser powers. According to the building constraints and theoretical analysis, the heat accumulates in the depth direction, so the point displacement occurs on the Z axis, which is perpendicular to the building scan direction. It can be seen from Figure 8a that the overall change in deformation history in point NT16 is increased first, which remains almost unchanged for a while and finally increases gradually after a certain amount of deformation. The metal powder absorbs heat and leads to the temperature increasing when the heat source is just near the initial point at the beginning, resulting in a change in the expansion coefficient. The built part that changes the expansion coefficient has a deformation on a single layer.

With time going by, the heat source gradually moves away from the point. Currently, the temperature remains almost unchanged due to the slow cooling, so the deformation remains substantially constant. When scanning for the second time, the heat source increasingly approaches from the end far away from the point. The material thermal cumulative effect gradually increases and finally reaches the peak. The maximum deformation reaches values of 0.015, 0.018, 0.021, and 0.026 for a laser power of 125, 150, 175, and 200 W, respectively.

As with the general trend of temperature distribution, the deformation gradually increases with the power increasing. Transient deformation of different powers at the same time can be known from Figure 8b. The amount of deformation is larger due to the metal material’s initial heating temperature when the laser power is lower. The place where the heat source does not pass is slightly deformed due to heat conduction and thermal expansion. However, when the laser power *P* = 200 W, the laser position is fixed at the midpoint of the first scanning. The overall displacement deformation on the midline decreases at the beginning and then increases. According to the material heat absorption and heat transfer, the powder is initially exposed to higher laser power, and the increase in temperature gradient makes the material easily deformed and expanded. When the units on the midline have been obtained preheating, the temperature gradient and the material deformation are correspondingly reduced. When the heat source moves to the midpoint for second scanning, the surrounding lower temperature and the higher power build a temperature gradient again. Material deformation increases gradually after thermal expansion.

#### 3.2.3. Temperature Distribution under Different Scanning Speeds

**Figure 9:**
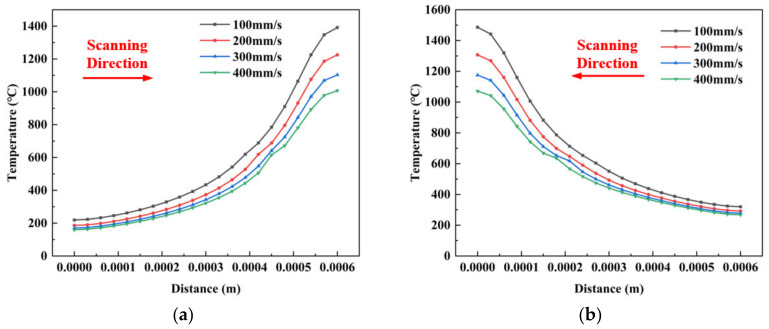
Different scanning speed temperature distributions at the end of two scans. (**a**) temperature distribution at the end of the first scans (**b**) temperature distribution at the end of the second scans.

Figure 9 shows the temperature profile for different speeds with *P* = 200 W and *D* = 0.2 mm. Figure 9a illustrates the whole temperature distribution on the midline between the two melt tracks when the heat source is at the end of the first analysis step. Figure 9b shows the same with (a) at the end of the second analysis step. According to the information in Figure 9, when *v* = 100 mm/s, the peak values are 1392 °C and 1486 °C. When *v* = 200 mm/s, the peak values are 1226 °C and 1307 °C. When *v* = 300 mm/s, the peak values are 1103 °C and 1175 °C. When *v* = 400 mm/s, the peak values are 1007 °C and 1071 °C. 

The temperature gradually reduces when the laser scanning speed increases. As the scanning speed indirectly affects the material’s energy absorption, metal powder absorbs higher energy for a short period, whose time for powder cooling and solidifying are also correspondingly reduced. The melt pool does not reach the vaporization point easily, which is built continuously at this time, and the surrounding powder will not be vaporized or blown away. The temperature will also decrease under this condition, but when the laser speed is too fast, the laser energy is reduced to be insufficient to melt more powder, causing the melting temperature gradient to increase. This condition produces a discontinuous melt pool and spheroidization, which seriously affects the building quality.

A slow scanning speed (*v* = 100 mm/s) results in a large liquid melt pool and splash. Being the same with changing laser power, the temperature is accumulated because of the first preheating and heat accumulation when the second laser scans through the powder layer. However, the temperature changes at a fast scanning speed and is not altered significantly. This is mainly due to the fact that every unit of material absorbed little heat when the scanning speed is fast. The reduction of thermal diffusion produces insignificant heat accumulation and leads to a low preheating temperature.

#### 3.2.4. Temperature Distribution under Different Spot Diameters

**Figure 10:**
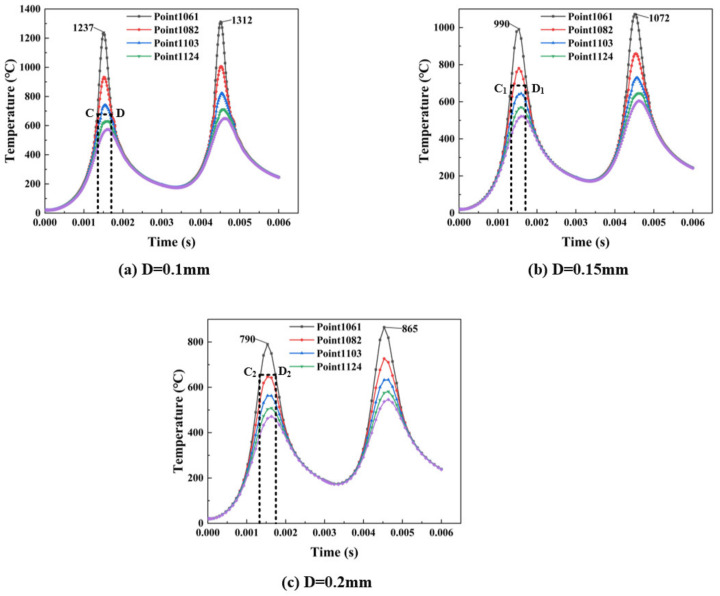
Temperature curve for different spot diameters with time changes (**a**) Temperature distribution when D = 0.1 mm (**b**) Temperature distribution when D = 0.15 mm (**c**) Temperature distribution when D = 0.2 mm.

Figure 10 shows the influence of changing the spot diameter on the temperature distribution. The scanning speed *v* = 200 mm/s and the laser power *P* = 150 W. It can be seen from Figure 10 that the temperature decreases with the spot diameter increasing. The distance between the two heat sources, concentrated in a circle with a diameter of 0.1 mm, is reduced when the spot diameter is small. Meanwhile, material absorbed the heat source energy and the temperature rose. The overlap ratio between the two scanning tracks is declining due to the small spot diameter. Pores produced during the process affect the building quality. The gradual increase in the spot diameter reduces the heat source distance and diffusion, resulting in a decrease in temperature. Owing to the previous preheating during the second laser scanning, the temperature gradually increases and shows an accumulation phenomenon. 

It can be seen from Figure 10 that it is slightly different from other process parameters. Figure 10a shows the temperature changes very sharply near the peak with a spot diameter of 0.1 mm. However, the temperature changes near the peak are slower when the spot diameter becomes wider (as shown in Figure 10b,c). As the spot diameter increases, the heat source is not concentrated, and the increase in heat diffusion and heat transfer of the material causes the surrounding temperature to rise. 

According to Figure 10a–c, the width of the melting zone should be indicated between C and D. Since the thermal conductivity of solids is much larger than that of powder, the temperature drops sharply at the boundary between the melting zone and the powder, forming melting zones with different widths, as shown in Figure 10. The distance between C_1_ and D_1_ is bigger than C and D with the spot diameter increasing, and the largest among them is between C_2_ and D_2_. At the same time, the peak maximum temperature gradually decreases and the decentralization of spots results in the heat transfer, diffusion capacity and width of melting zone increasing. It is not only spot diameters that affect the width; different laser powers and scanning speeds can also have different widths of melting zone.

## 4. Conclusions

This paper presents a finite element simulation model for the distribution of temperature and stress consideration of thermo-mechanical coupling in the selective laser melting of aluminum alloy powder for process monitoring and quality control. Based on this model, the influence of the process parameters in SLM on the distribution of temperature, thermal stress and deformation are studied. The following conclusions can be drawn.

1. Under the same process parameters of the laser, there is a large temperature gradient during the scanning process due to the effects of the heat accumulation and heat conduction. Furthermore, the temperature and size of the melt pool are significantly larger than the previous one because of the heat accumulation when the laser scans from the first pass to the second.
2. During the movement of the laser heat source, the temperature near the heat source gradually increases due to latent heat and conduction, and gradually decreases away from the heat source.
3. The temperature of the melted powder particles accumulates and increases with the increasing of the laser power as energy is absorbed from the heat source. However, the temperature gradient of the material will reduce with increasing laser power, and the stress will decrease correspondingly. Furthermore, the material powder, such as the Al6063 powder, will remelt and form coarse grains with increasing laser power, which can affect the mechanical properties of the part. In contrast, when the power is too low, the melt pool cannot be formed because the melting point of the powder cannot be reached.
4. With an increased scanning speed, the temperature of the melting pool will reduce because the energy absorbed by the powder decreases. Conversely, the temperature of the melting pool will increase with the decreasing scanning speed.
5. When the spot diameter increases, the temperature melting pool will decrease because the heat diffuses to the surrounding environment due to the unconcentrated heat source. Meanwhile, different spot diameters will form different widths of the melting zones, since the thermal conductivity of the solid is much larger than the powder and the temperature at the boundary of the melting zone and the powder drops sharply.
6. The model established in this paper is mainly to analyze the transient temperature field, stress field distribution and melt pool morphology prediction under different building parameters. In the specific applications, the size of the model can be appropriately increased or reduced according to different building conditions. For the powder materials in the model, if the powder material is different, such as the titanium alloy which has high temperature resistance, high strength and good corrosion resistance, it is necessary to increase the scanning power or reduce the scanning speed and other building parameters for simulation analysis. For the verification of residual stress and strain, the follow-up research will be to build a real-time monitoring platform based on a multi-sensor and intelligent detection system.

## Figures and Tables

**Table 1:** Thermal property parameters of Al6063 and 1045 steel [22,23].

Material	Temperature (°C)	Density (Kg/m^3^)	Thermal Conductivity (W/(°C·m))	Specific Heat Capacity (J/(Kg °C))
Al6063	20	2700	119	900
	100	2700	121	921
	200	2700	126	1005
	300	2700	130	1047
	400	2700	135	1089
	2000	2700	145	1129
1045 steel	20	7890	47.6	472
	100	7890	43.5	480
	200	7890	40.4	498
	300	7890	38.1	560
	400	7890	36.0	589
	2000	7890	24.0	602

**Table 2:** Laser processing parameters.

Simulation Groups	Initial Temperature *T*_0_ (°C)	Spot Diameter *D* (mm)	Scanning Speed *v* (mm/s)	Laser Power *P* (W)
1–4	20	0.1	200	125, 150, 175, 200
5–8	20	0.1	100, 200, 300, 400	150
9–11	20	0.1, 0.15, 0.2	200	150

## Data Availability

The data presented in this study are available in the manuscript.

## References

[1] Li W., Huang H., Tian Y., Zhao Z. (2015). Nonlinear analysis on thermal behavior of charring materials with surface ablation. Int. J. Heat Mass Transf..

[2] Li W., Huang H., Tian Y., Zhao Z. (2015). A nonlinear pyrolysis layer model for analyzing thermal behavior of charring ablator. Int. J. Therm. Sci..

[3] Li W., Huang H., Ai B., Zhang Z. (2016). On the novel designs of charring composites for thermal protection application in reentry vehicles. Appl. Therm. Eng..

[4] Zhang Y., Hong G.S., Ye D., Zhu K., Fuh J.Y. (2018). Extraction and evaluation of melt pool, plume and spatter information for powder-bed fusion AM process monitoring. Mater. Des..

[5] Zhang Y., Fuh J.Y., Ye D., Hong G.S. (2019). In-situ monitoring of laser-based PBF via off-axis vision and image processing approaches. Addit. Manuf..

[6] Ye D., Zhu K., Fuh J.Y.H., Zhang Y., Soon H.G. (2019). The investigation of plume and spatter signatures on melted states in selective laser melting. Opt. Laser Technol..

[7] Grünberger T., Domröse R. (2015). Direct metal laser sintering. Laser Tech. J..

[8] Grasso M., Demir A.G., Previtali B., Colosimo B.M. (2018). In situ monitoring of selective laser melting of zinc powder via infrared imaging of the process plume. Robot. Comput. Integr. Manuf..

[9] Gökhan Demir A., De Giorgi C., Previtali B. (2018). Design and Implementation of a Multisensor Coaxial Monitoring System With Correction Strategies for Selective Laser Melting of a Maraging Steel. J. Manuf. Sci. Eng..

[10] Kollossov S., Boillat E., Glardon R., Fisher Y., Locher M. (2004). 3D FE simulation for temperature evolution in the selective laser sintering process. Int. J. Mach. Tools Manuf..

[11] Patil R.B., Yadava V. (2007). Finite element analysis of temperature distribution in single metallic powder layer during metal laser sintering. Int. J. Mach. Tools Manuf..

[12] Cao L. (2019). Numerical simulation of the impact of laying powder on selective laser melting single-pass formation. Int. J. Heat Mass Transf..

[13] Ding X., Wang L. (2017). Heat transfer and fluid flow of molten pool during selective laser melting of AlSi10Mg powder: Simulation and experiment. J. Manuf. Process..

[14] Li Y., Zhou K., Tor S.B., Chua C.K., Leong K.F. (2017). Heat transfer and phase transition in the selective laser melting process. Int. J. Heat Mass Transf..

[15] Loh L.E., Chua C.K., Yeong W.Y., Song J., Mapar M., Sing S.L., Liu Z.H., Zhang D.Q. (2015). Numerical investigation and an effective modelling on the Selective Laser Melting (SLM) process with aluminium alloy 6061. Int. J. Heat Mass Transf..

[16] Huang Y., Yang L.J., Du X.Z., Yang Y.P. (2016). Finite element analysis of thermal behavior of metal powder during selective laser melting. Int. J. Therm. Sci..

[17] Matsumoto M., Shiomi M., Osakada K., Abe F. (2002). Finite element analysis of single layer forming on metallic powder bed in rapid prototyping by selective laser processing. Int. J. Mach. Tools Manuf..

[18] Tawfik S.M., Nasr M.N., El Gamal H.A. (2019). Finite element modelling for part distortion calculation in selective laser melting. Alex. Eng. J..

[19] Liu H. (2014). Numerical analysis of thermal stress and deformation in multi-layer laser metal deposition process. Diss. Theses Gradworks.

[20] Cheng B., Shrestha S., Chou K. (2016). Stress and deformation evaluations of scanning strategy effect in selective laser melting. Addit. Manuf..

[21] Sumin Sih S., Barlow J.W. (1994). Measurement and prediction of the thermal conductivity of powders at high temperature. Austion.

[22] Montenegro C., Abolghasem S., Osorio-Pinzon J.C., Casas-Rodriguez J.P. (2020). Microstructure prediction in low and high strain deformation of Al6063 using artificial neural network and finite element simulation. Int. J. Adv. Manuf. Technol..

[23] Wang H., Yang H.B. (2013). 6063 Aluminum Alloy Online Quenching Surface Heat Transfer Coefficient and the Temperature Field Simulation. Appl. Mech. Mater..

[24] Jiang X.F., Meng X.C., Song R.W., Yuan X.G., Ye M. (2015). Study on the effects of temperature field of material state change in the SLM process. Appl. Laser.

[25] Carslawhs J. (1967). Conduction of Heat in Solids.

[26] Shipley H., McDonnell D., Culleton M., Coull R., Lupoi R., O’Donnell G., Trimble D. (2018). Optimisation of process parameters to address fundamental challenges during selective laser melting of Ti-6Al-4V: A review. Int. J. Mach. Tools Manuf..

[27] Alimardani M., Toyserkani E., Huissoon J.P., Paul C.P. (2009). On the delamination and crack formation in a thin wall fabricated using laser solid freeform fabrication process: An experimental-numerical investigation. Opt. Lasers Eng..

